# Co-Circulation of 72bp Duplication Group A and 60bp Duplication Group B Respiratory Syncytial Virus (RSV) Strains in Riyadh, Saudi Arabia during 2014

**DOI:** 10.1371/journal.pone.0166145

**Published:** 2016-11-11

**Authors:** Anwar Ahmed, Shakir H. Haider, Shama Parveen, Mohammed Arshad, Hytham A. Alsenaidy, Alawi Omar Baaboud, Khalid Fahad Mobaireek, Muslim Mohammed AlSaadi, Abdulrahman M. Alsenaidy, Wayne Sullender

**Affiliations:** 1 Protein Research Chair, Department of Biochemistry, College of Science, King Saud University, Riyadh, Saudi Arabia; 2 Centre of Excellence in Biotechnology Research, Department of Biochemistry, College of Science, King Saud University, Riyadh, Saudi Arabia; 3 Centre for Interdisciplinary Research in Basic Sciences, Jamia Millia Islamia, New Delhi, India; 4 College of Medicine, Shaqra University, Shaqra, Saudi Arabia; 5 Pediatric Emergency Department, Children’s Hospital, King Fahad Medical City, Riyadh, Saudi Arabia; 6 Department of Pediatrics, College of Medicine, King Khalid University Hospital, King Saud University, Riyadh, Saudi Arabia; 7 School of Medicine, Center for Global Health, Colorado School of Public Health, Aurora, Colorado, United States of America; University of Tennessee Health Science Center, UNITED STATES

## Abstract

Respiratory syncytial virus (RSV) is an important viral pathogen of acute respiratory tract infection (ARI). Limited data are available on molecular epidemiology of RSV from Saudi Arabia. A total of 130 nasopharyngeal aspirates were collected from children less than 5 years of age with ARI symptoms attending the Emergency Department at King Khalid University Hospital and King Fahad Medical City, Riyadh, Saudi Arabia between October and December, 2014. RSV was identified in the 26% of the hospitalized children by reverse transcriptase PCR. Group A RSV (77%) predominated during the study as compared to group B RSV (23%). The phylogenetic analysis of 28 study strains clustered group A RSV in NA1 and ON1 genotypes and group B viruses in BA (BA9) genotype. Interestingly, 26% of the positive samples clustered in genotypes with duplication in the G protein gene (ON1 for group A and BA for group B). Both the genotypes showed enhanced O-linked glycosylation in the duplicated region, with 10 and 2 additional sites in ON1 and BA respectively. Selection pressure analysis revealed purifying selection in both the ON1 and BA genotypes. One codon each in the ON1 (position 274) and BA genotypes (position 219) were positively selected and had high entropy values indicating variations at these amino acid positions. This is the first report describing the presence of ON1 genotype and the first report on co-circulation of two different genotypes of RSV with duplication in the G protein gene from Saudi Arabia. The clinical implications of the simultaneous occurrence of genotypes with duplication in G protein gene in a given population especially in the concurrent infections should be investigated in future. Further, the ongoing surveillance of RSV in this region will reveal the evolutionary trajectory of these two genotypes with duplication in G protein gene from largest country in the Middle East.

## Introduction

RSV is an important viral pathogen of acute respiratory tract infection (ARI). RSV causes around 33.8 million infections and 2.8 to 4.3 million hospital admissions with 66000 to 199000 deaths annually across the globe [1]. RSV has a single stranded negative sense RNA genome of approximately 15.2 Kb. The surface proteins of the virus include the G (glycoprotein), F (fusion protein) and SH (small hydrophobic protein). The G protein is type II glycoprotein that is involved in attachment of virion to the host cell. It is rich in serine and threonine residues and is therefore highly glycosylated. The G protein is a neutralizing antigen and thus a vaccine candidate. The second hypervariable region of the G protein is a hotspot for mutations and has been analyzed in the epidemiological studies [2,3,4]. RSV has been classified into two groups (group A and B) on the basis of genetic and antigenic heterogeneity. Group A strains have been categorized in to various genotypes (GA1-GA7, SAA1, SAA2, NA1, NA2 and ON1) and group B into following genotypes (GB1-GB6, SAB1-SAB4 and BA). The BA genotype is further divided in to sub groups (BA1 to BA12) [5,6,7,8,9,10,11].

Genetic variations occur in RSV due to mutation especially in the second hypervariable region of the G protein gene. Two such cases of drastic modifications occurred in RSV genome in 1999 and 2011. In 1999, noteworthy 60bp duplication occurred in the second hypervariable region of the G protein gene of group B RSV in Argentina [12]. Subsequently, remarkable 72bp duplication occurred in the same region of RSV genome in group A viruses in Canada in 2011 [5]. It has been postulated that this duplication of part of the gene occurred probably due to “backtracking” of the RNA dependent RNA polymerase [5,13]. Later on, these genotypes with duplication spread rapidly to different geographical regions probably due to immunologically naive populations [3].

The ON1 genotype has been reported from 21 countries [11] including Thailand [9], China [10], Canada [5], Japan [14,15], USA [16], Kenya [17], South Africa [18], Malaysia [7], Latvia [19], India [20], Korea [21,22], Italy [23], Cyprus [24], Germany [25,26], Spain [2]. Similarly, the BA viruses were also described from various countries including Brazil [27], Belgium [28,29], Spain [30], Argentina [12,31,32,33], India [34,35], South Africa [36], Japan [37], China [38], Kenya [39,40], Saudi Arabia [41], Italy, Germany, Mexico, Argentina, USA [42] and Japan [43].

The present study was conceptualized to characterize the circulating strains of group A and B RSV from Riyadh, Saudi Arabia during 2014. The central benchmark of the present study is that it is the first description of the co-circulation of ON1 and BA genotypes of RSV with duplication in G protein gene from the largest country in Middle East. Determination of the genetic composition of RSV strains circulating in different geographical regions will be important during evaluation of initial vaccine trials.

## Materials and Methods

### Ethics statement

Institutional Review Boards of College of Medicine, King Saud University (approval no. E-14-1155) and King Fahad Medical City (approval no. 14–279), approved the study protocol. Written informed consent in English/Arabic was obtained from the parents/guardian prior to enrollment of children in the study.

### Sample collection

The study was carried out at two hospitals, King Khalid University Hospital (KKUH) and King Fahd Medical City (KFMC), Riyadh, Saudi Arabia. Riyadh, the capital of Saudi Arabia is situated in the Central region. KKUH is an 800 bed facility with all general and subspecialty medical services. The hospital provides primary and secondary care services for patients from Northern Riyadh region. KFMC is a more than 1000 bed facility and is the largest medical hospital in the Middle East which comprises of 8 hospitals and specialty Centres. The Children’s hospital at KFMC and Pediatrics Department at KKUH were the sites of sample collection.

Children less than or equal to five years of age attending Emergency Department (ED) or admitted to the ward with ARI symptoms [44] at KKUH and KFMC from October to December 2014, were enrolled for the study. Children with ARI were examined by the pediatrician and the clinical information of the patients was collected in proformas. Nasopharyngeal aspirates (NPAs) were collected from the patients by a trained nurse/technician in 1ml viral collection tubes (UTM Copan, Brescia, Italy). The samples were transported to the laboratory on ice within 2–4 hours. The samples were processed and stored at -80C till RNA was extracted.

### RNA extraction and cDNA synthesis

The samples were vortexed for 1 min and viral RNA was extracted from 500 μl of sample using the RNeasy Mini Kit (Qiagen, CA, USA) according to the manufacturer’s instructions. Reverse transcription was performed using Superscript II reverse transcriptase (Invitrogen Life technologies, CA, USA) according to the manufacturer’s instructions.

### RT-PCR for detection of RSV

The second hypervariable region of the G protein gene of RSV was the target for amplification. The external PCR was performed using published primers and cycling conditions [45]. The external amplicons (602bp to 726bp) was diluted and used for the nested PCR. The semi-nested PCR was carried out using protocol described earlier [34,45]. The amplicons were run on 2% agarose gel and visualized with GelDoc-It2 Imager (Ultra-Violet Products Ltd, Cambridge, UK). The size of the nested amplicons were 450/585 bp, 645 bp and 522 bp for RSV-A/B, BA and ON1genotypes, respectively.

### DNA sequencing of G protein gene of RSV

The amplicons of nested PCR were extracted from the gel using QIAquick Gel Extraction Kit (QIAGEN, CA, USA), as per manufacturer’s instructions. The amplicons were sequenced commercially from Macrogen Inc, Korea, in forward and reverse direction using the nested primers. The group A and group B RSV sequences were confirmed by BLAST. The forward and reverse sequences were aligned and manually edited in BioEdit software version 7.2.5 [46].

### Phylogenetic analysis of the G protein gene sequences

The RSV sequences of both the group A and B RSV were downloaded from Genbank. Multiple sequence alignment was done with ClustalW of the BioEdit software version 7.2.5 [46]. Phylogenetic trees were constructed using Maximum Likelihood method in MEGA 6 software [47]. The robustness of the tree was accessed with 1000 replicas. The genetic distances were calculated using Kimura 2 method of nucleotide substitution. The following prototype strains were used in the study: GenBank Accession Number AB470478 for NA1 genotype of RSV A, Accession Number JN257694 for ON1 genotype of RSV A and Accession Number AY333364 for BA genotype of RSV B.

### Analysis of deduced amino acid sequences and mutations

The amino acid sequences of the second hypervariable region of the G protein were predicted for both the group of RSV with standard genetic code. The mutations were described for group A and group B RSV with respect to their prototype strains.

### Selection pressures analysis

Selection pressure in the second hyper variable region of the G protein gene was studied using Datamonkey server (http://www.datamonkey.org/) [48]. The non-synonymous to synonymous mutations ratio (dN/dS) was calculated using two different methods, Single Likelihood Ancestor Counting (SLAC) and Fixed Effect Likelihood (FEL) by using HKY85, F81 and REV model of nucleotide substitution. The positively selected sites were considered as the sites under weak selection pressure by at least two different methods with p≤0.2 (p = 0.05, 0.1, 0.15, 0.2).

### Entropy analysis

Shanon entropy analysis was carried out in BioEdit (ver. 7.2.5) to analyze the amino acid variation in the G protein gene. The calculated entropy values were exported and plotted in Microsoft Excel to generate the entropy plot. The entropy values ranged from 0 to 0.93 with Shanon entropy threshold value of 0.2. The amino acids with value less than 0.2 were considered conserved whereas the values more than 0.2 were considered as the variable sites as described earlier [3].

### N- and O-linked glycosylation sites

The potential N-glycosylation sites (NXT, where X is not a proline) and O-glycosylation sites of the G proteins were predicted using NetNGlyc1.0 (http://www.cbs.dtu.dk/services/NetNGlyc) and NetOGlyc3.1 (http://www.cbs.dtu.dk/services/NetOGlyc) [49].

## Results

### Patient characteristics

One hundred and thirty clinical samples were collected from the children with ARI symptoms during October to December 2014. Fifty five samples were collected from the King Khalid University Hospital (KKUH) and 75 samples from King Fahd Medical City (KFMC), Riyadh, Saudi Arabia. The clinical manifestations of the study patients are given in Table 1. The mean age of the patients was 1.2 years (SD±1.06 years) and the male to female ratio was 1.4. The patients presented with the symptoms of fever, nasal discharge, cough and sore throat (Table 1).

**Table 1:** Clinical manifestations of the study patients.

Age group	No. of cases	Gender		Symptoms (%)			
		Boys	Girls	Fever	Nasal Discharge	Cough	Sore throat
0–6 months	41	25	16	41 (100)	41 (100)	39 (95)	38 (93)
>6–12 months	38	22	16	38 (100)	38 (100)	37(97)	37(97)
>1–2 years	24	13	11	22(92)	21(88)	20(83)	20(83)
>2–3 years	15	10	5	13(87)	12(80)	12(80)	11(73)
>3–5 years	12	6	6	10(83)	9(75)	9(75)	8(67)
**Total**	**130**	**76**	**54**				

### RSV prevalence

All the samples were tested for RSV by RT-PCR. RSV was detected in 35 samples (27%) of the 130 samples tested. Group A RSV was identified in 27 (77%) and group B RSV in 8 (23%) of the positive samples. The age wise distribution of the RSV positive cases is given in Table 2. Maximum number of positive RSV cases were observed in the 0–6 months age group. Co-infection with both the subgroups of RSV was detected in 2 (5.7%) of the RSV positive samples. Thirty one percent of the samples (17/55) were positive for RSV from KKUH hospital and 24% (18/75) from KFMC hospital.

**Table 2:** Age wise distribution of the RSV positive cases.

Age group	Total no. of cases	No. of RSV positive cases	RSV A	RSV B
0–6 months	41	11	8	3
>6–12 months	38	9	7	2
>1–2 years	24	7	5	2
>2–3 years	15	5	5	0
>3–5 years	12	3	2	1
**Total**	**130**	**35**	**27**	**8**

### DNA sequencing and GenBank Accession Numbers

Nucleotide sequences of second hyper variable region of the G protein gene were determined for 23 group A and 5 group B strains. All the 28 study sequences were deposited in the GenBank with the following Accession Numbers: KU726066-KU726088 for group A RSV and KU726061-KU726065 for group B RSV.

### Phylogenetic analysis

#### RSV group A strains

**Fig 1:**
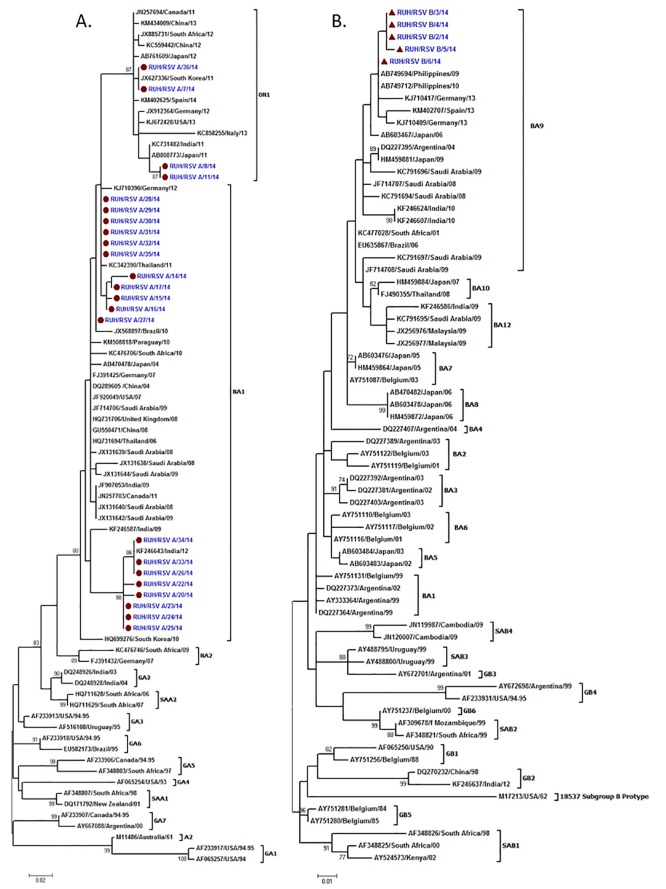
Phylogenetic trees for hRSV group A (A) and group B (B). The nucleotide sequences from the second variable region of the G protein gene of the study RSVA strains are indicated by solid circles and RSVB by solid triangles. The nucleotide sequences were aligned with the CLUSTAL W program, and phylogenetic trees were constructed by Maximum Likelihood method using MEGA6 software. Bootstrap values greater than 70% are shown at the branch nodes.

Seventy eight RSV sequences including 23 study sequences were used for the phylogenetic analysis (S1 Table). A 216bp region corresponding to 679-894bp of G protein gene in NA1 prototype strain and 288bp region corresponding to 679-966bp of G protein gene in ON1 prototype strain of RSV was used for the alignment. Nineteen study sequences clustered in NA1 and 4 sequences in ON1 genotype (Fig 1A). The nucleotide and amino acid distance between the NA1 study strains and prototype NA1 strain was 1.4% to 6.1% and 1.4% to 15.2% respectively. The nucleotide and amino acid distance among the study strains was upto 6.7% and upto 16.8% respectively. On comparing all Saudi strains the nucleotide and amino acid distance was upto 5.6% and 15.2%, respectively. The nucleotide and amino acid distance between the ON1 study strains and prototype ON1 strain was 0.3% to 2.5% and upto 4.3% respectively. The nucleotide and amino acid distance among the study strains was upto 2.8% and upto 4.3% respectively.

#### RSV group B strains

Seventy RSV sequences including 5 study sequences were used for the phylogenetic analysis (S2 Table). A 312bp region corresponding to 637bp to 948bp of the G protein gene of BA prototype strain of RSV was used for the alignment. All the 5 study sequences grouped in BA9 subgroup of the BA genotype (Fig 1B). The study strains showed nucleotide distance of 4.2 to 5.3% and amino acid distance of 7.3% as compared to the BA prototype strain. The study strains showed 1% nucleotide distances and amino acid sequence of all study strains were identical. The study strains had nucleotide distance of up to 1.4 to 8.4% and amino acid distance of 1% to 15.1% as compared to other Saudi strains. Interestingly, 26% of the positive samples clustered in genotypes with duplication in the G protein gene (ON1 for group A and BA for group B).

### Mutational analysis

**Fig 2:**
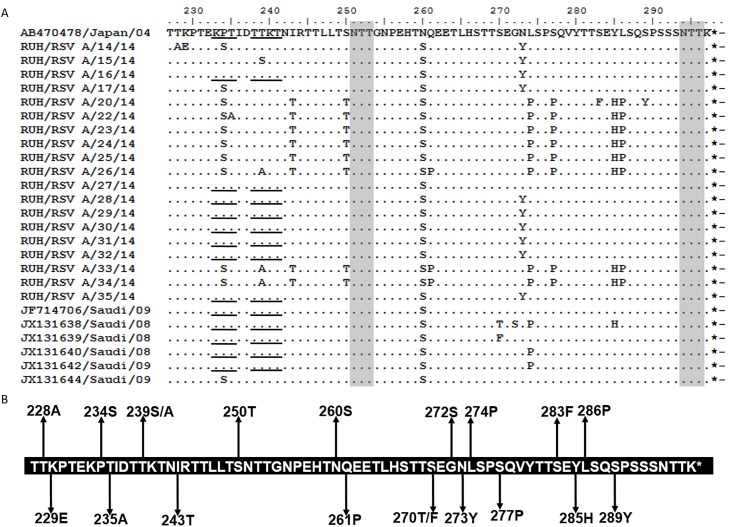
**(A) Deduced amino acid alignment and mutations in the second hyper-variable region of G protein of the NA1 genotype.** The figure includes alignment of Saudi NA1 strains with the prototype strain from Japan (AB470478). The amino acids sequence alignment corresponds to 227–297 amino acids of the prototype strain. Identical residues are indicated by dashes. Stop codons are indicated by asterisks. Potential N-glycosylation sites (NXT, where X is not proline) are indicated by grey shading. The potential sites for extensive O-glycosylation KPX - - - TTKX motifs are underlined. **(B) The amino acid sequence of NA1 genotype showing mutations in the Saudi strains.** The sequence corresponds to the 227–297 amino acids of the NA1 prototype strain. Changes at amino acid positions in Saudi strains are shown by arrows.

The predicted protein length of the group A and group B study sequences were compared with the respective prototype strains. All the NA1 study strains were predicted to encode G protein of 297 amino acids which is similar to the prototype strain from Japan (Fig 2A). Mutational analysis revealed that all the Saudi NA1 strains (including study and reported sequences) had Serine at 260 position compared to Aspargine in prototype sequence. They all have TGA as stop codon that is similar to the prototype strain. A total of 18 mutations were identified in the NA1 sequences from Saudi as compared to prototype strain (Fig 2B). Further, sixteen mutations were identified in study sequences while mutations at 270 (S270T/F) and 272 (G272S) were reported in earlier NA1 sequences from Saudi which are not observed in the study sequences. A total of 12 new mutations were observed in the study sequences as compared to the sequences reported earlier from Saudi Arabia. Two unique mutations corresponding to T228A and K229E were observed in one of the study sequences (RUH/RSV A/14/14) and the other study sequence (RUH/RSV A/20/14) also showed unique mutation at S283F and S289Y positions. One more study sequence (RUH/RSV A/22/14) had T235A mutation. The study sequence (RUH/RSV A/15/14) had T239S substitution while three others (RUH/RSV A/26/14) (RUH/RSV A/33/14) (RUH/RSV A/34/14) sequences showedT239A substitution.

**Fig 3:**
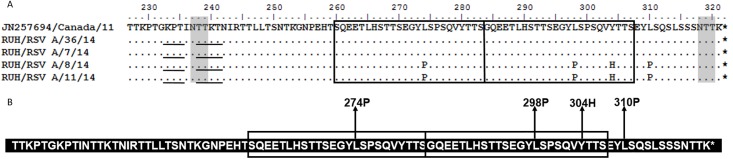
**(A) Deduced amino acid alignment and mutations in the second variable region of G protein of the ON1 genotype.** The figure includes alignment of study ON1 strains with the prototype strain from Canada (JN257694). The amino acids sequence alignment corresponds to 227–321 amino acids of the prototype strain. Identical residues are indicated by dashes. The two copies of the duplicated 24-amino-acid region in group ON1 strains are indicated by rectangular boxes. Stop codons are indicated by asterisks. Potential N-glycosylation sites (NXT, where X is not proline) are indicated by grey shading. The potential sites for extensive O-glycosylation KPX - - - TTKX motifs are underlined. **(B) The amino acid sequence of ON1 genotype showing mutations in the study strains.** The sequence corresponds to the 227–321 amino acids of the ON1 prototype strain. The rectangular boxes represent the analogous 24 amino acid region followed by duplicated 24 amino acid region in ON1 strains. Changes at amino acid positions in study strains are shown by arrows.

Similarly, protein length of all the ON1 study strains was predicted to be 321 amino acids which are similar to the corresponding prototype strain from Canada (Fig 3A). Four mutations were identified in the ON1 sequences from Saudi as compared to the prototype strain. Two of the samples (RUH/RSVA/7/14 and RUH/RSVA/36/14) had protein sequences identical to prototype strain in region from 227 to 321 amino acids (Fig 3B). The amino acid substitution L274P was seen in two of the study samples in analogous region. Corresponding L298P mutation was also seen in the duplicated 24 amino acid sequence followed by Y304H substitution. Proline, which was a substitution in the analogous and duplicated region, was also seen outside the duplicate region substituting L310P.

**Fig 4:**
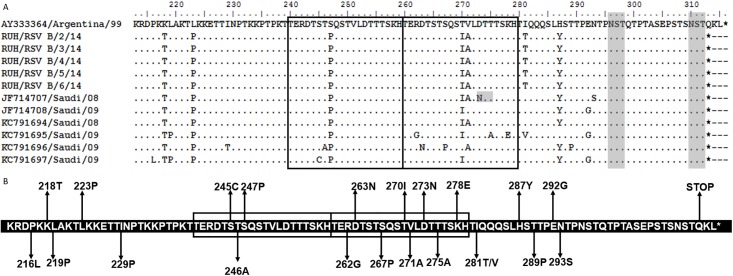
**(A) Deduced amino acid alignment and mutations in the second variable region of G protein of the BA genotype.** The figure includes alignment of Saudi BA strains with the prototype BA strain from Argentina (AY333364). The amino acids sequence alignment corresponds to 213–315 amino acids of the prototype strain. Identical residues are indicated by dashes. The two copies of the duplicated 20-amino-acid region in group BA strains are indicated by rectangular boxes. Stop codons are indicated by asterisks. Potential N-glycosylation sites (NXT, where X is not proline) are indicated by grey shading. **(B) The amino acid sequence of the BA genotype showing mutations in the Saudi strains.** The sequence corresponds to the 227–297 amino acids of the BA prototype strain. The rectangular boxes represent the analogous 20 amino acid region followed by duplicated 20 amino acid region in BA RSV strains. Changes at amino acid positions in Saudi strains are shown by arrows.

All the BA study sequences had protein length of 312 amino acids with reference to the prototype strain from Argentina (315 amino acids) (Fig 4A). A total of 21 mutations were identified in the BA sequences from Saudi as compared to the prototype strain (Fig 4B). Seven mutations were identified in the study sequences and these mutations were also found in the earlier reported sequences from Saudi except I281T which is not found in earlier sequences. Instead one of the sequences (KC791695) has V at 281 position. Fifteen of the mutations reported in earlier sequences were not observed in the study sequences. All the Saudi strains had K218T, L223P, S247P, T270I and H287Y substitution except in the sequence KC791696 where no T270I and the sequence KC791695 where no H287Y substitution was found. All the sequences have TGA as stop codon that is similar to the prototype strain.

### Selection pressure analysis

The selection pressure analysis was done for both the groups of RSV. The RSV sequences that were used for selection pressure analysis are given in (S1 and S2 Tables). The selection pressure analysis of the NA1 genotype strains revealed low ratio of dN/dS (0.82–1.04) by using different methods suggesting that the codon positions were relatively conserved (Table 3). This data set consisted of the NA1 genotype sequences (n = 42) gave a dN/dS ratio of 0.992, 0.823, and 1.042 by SLAC method using HKY85, F81 and REV methods of nucleotide substitution, respectively. The SLAC analysis further showed positive selection at 1 codon (position 274) by HKY85 and REV method of nucleotide substitution. The SLAC analysis also revealed upto 5 negatively selected sites in this data set. The FEL analysis revealed 1 to 9 negatively selected sites and 4 weakly positively selected sites (codon number 234, 239, 260, 273 and 274).

**Table 3:** Selection pressure analysis of the RSV NA1 genotype of the second hypervariable region of G protein gene using SLAC and FEL method.

NA1 genotype															
Nucleotide substitution method →	SLAC									FEL					
	HKY85			F81			REV			HYK85		F81		REV	
P value↓	dN/dS	selected sites		dN/dS	selected sites		dN/dS	selected sites		selected sites		selected sites		selected sites	
		+ve	-ve		+ve	-ve		+ve	-ve	+ve	-ve	+ve	-ve	+ve	-ve
0.05	0.992	-	-	0.823	-	-	1.042	-	-	-	1	-	3	-	2
0.1	0.992	-	-	0.823	-	2	1.042	-	-	-	4	-	6	-	4
0.15	0.992	-	2	0.823	-	5	1.042	-	2	-	6	1 (234)	7	-	5
0.2	0.992	1 (274)	2	0.823	-	5	1.042	1 (274)	1	1 (234	7	2 (234, 239)	9	4 (239, 260, 273, 274)	7

The selection pressure analysis of ON1 strains revealed low ratio of dN/dS (0.78–0.88) by using different methods suggesting that the codon positions are relatively conserved (Table 4). The data set consisted of the RSVA ON1 genotype sequences (n = 16) gave a dN/dS ratio of 0.87, 0.78, and 0.88 by SLAC method using HKY85, F81 and REV methods of nucleotide substitution. The SLAC analysis further showed no positive selection in this data set, but one negatively selected site was observed. The FEL analysis revealed 1–6 negatively selected sites and 1 weakly positively selected site (codon number 274).

**Table 4:** Selection pressure analysis of the RSV ON1 genotype of the second hypervariable region of G protein gene using SLAC and FEL method.

ON1 genotype															
Nucleotide substitution method →	SLAC									FEL					
	HKY85			F81			REV			HYK85		F81		REV	
P value ↓	dN/dS	selected sites		dN/dS	selected sites		dN/dS	selected sites		selected sites		selected sites		selected sites	
		+ve	-ve		+ve	-ve		+ve	-ve	+ve	-ve	+ve	-ve	+ve	-ve
0.05	0.868	-	-	0.778	-	-	0.880	-	-	-	-	-	-	-	-
0.1	0.868	-	-	0.778	-	-	0.880	-	-	-	-	-	2	-	-
0.15	0.868	-	-	0.778	-	1	0.880	-	-	-	5	-	4	-	-
0.2	0.868	-	1	0.778	-	1	0.880	-	1	1 (274)	5	1 (274)	6	-	2

The selection pressure analysis of the data set comprising only the sequences of BA genotype (n = 50) also showed low dN/dS ratio (0.476) by SLAC analysis (Table 5). Further, this data set showed 2 positively selected sites (codon 219 and 267) sites by SLAC analysis using HKY85, F81 and REV method of nucleotide substitution. This data set showed 3–9 negatively selected sites by SLAC analysis. The FEL analysis showed 5–21 negative selection sites using HKY85, F81 and REV method of nucleotide substitution. Further, the FEL analysis revealed 1–7 positively selected sites (codons 219, 246, 247, 251, 267, 282, 289) by different methods. One particular codon at position 219 was found to be positively selected by both SLAC and REL analysis using two different methods of nucleotide substitution.

**Table 5:** Selection pressure analysis of the RSV BA genotype of the second hypervariable region of G protein gene using SLAC and FEL method.

BA genotype															
Nucleotide substitution method →	SLAC									FEL					
	HKY85			F81			REV			HYK85		F81		REV	
P value ↓	dN/dS	selected sites		dN/dS	selected sites		dN/dS	selected sites		selected sites		selected sites		selected sites	
		+ve	-ve		+ve	-ve		+ve	-ve	+ve	-ve	+ve	-ve	+ve	-ve
0.05	0.476	-	3	0.441	-	4	0.465	-	3	-	5	-	5	-	5
0.1	0.476	1 (219)	4	0.441	1 (267)	5	0.465	1 (219)	4	-	15	1(267)	9	-	13
0.15	0.476	1 (219)	4	0.441	1 (267)	5	0.465	1 (219)	4	3 (219, 251, 267)	19	4 (219, 247, 251, 267)	15	-	18
0.2	0.476	2 (219, 267)	5	0.441	1 (267)	9	0.465	2 (219, 267)	5	-	19	7 (219, 246, 247, 251, 267, 282, 289)	21	1 (219)	19

### Entropy analysis

**Fig 5:**
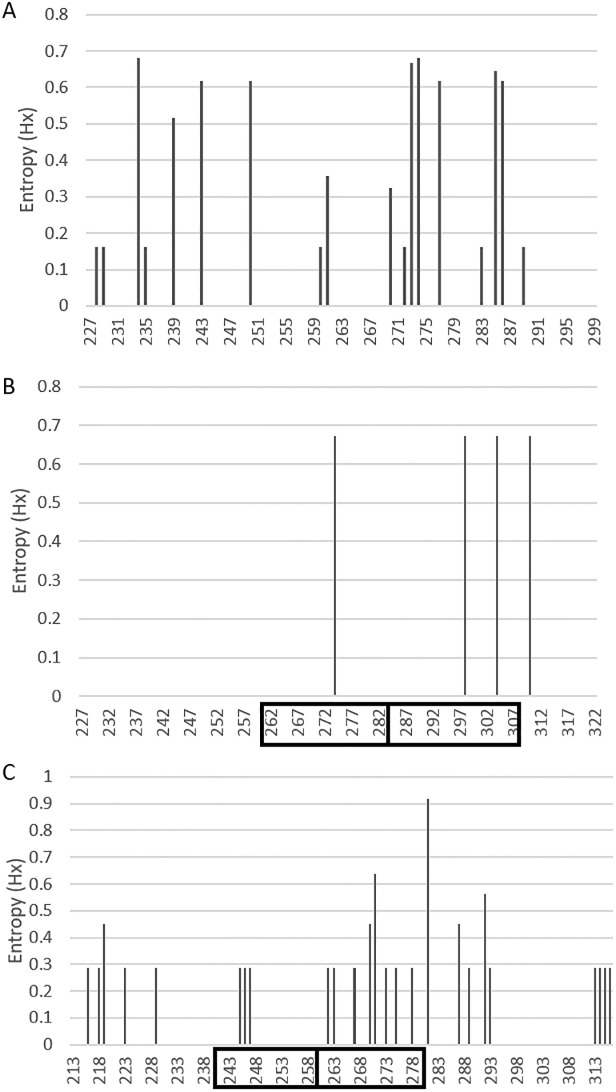
Shannon entropy plots of deduced amino acid sequences of the second hypervariable region of the G protein. The data set includes **(A)** RSV-A: NA1 genotype, n = 26 **(B)** ON1 genotype, n = 05 and **(C)** RSV-B: BA genotype, n = 11 of Saudi strains with their respective prototype strains. The entropy plots show the amino acid variability in the second hypervariable region of the G protein gene determined by BioEdit software. The threshold value was set at 0.2. Amino acid sites with entropy values <0.2 are considered conserved and values >0.2 are considered variable.

Shannon entropy analysis of the second hyper variable region of the G protein gene was carried out for all the three genotypes of the Saudi strains. The data set (n = 26) used for the entropy analysis of the NA1 genotype included the prototype sequence, 19 study sequences and 6 Saudi sequences that were reported earlier [50] (S1 Table). The analysis of NA1 genotype revealed that the 11 variable sites (position 234, 239, 243, 250, 261, 270, 273, 274, 277, 285 and 286) that were distributed throughout the sequence of G protein (Fig 5A). Eight (position 234, 243, 250, 273, 274, 277, 285 and 286) of these variable sites had entropy value of more than 0.6. Two different amino acids at position 234 and 274 were most variable in the NA1 genotype with entropy of value 0.68.

The data set (n = 5) for the ON1 genotype (S1 Table) included the prototype sequence and 4 study sequences (Fig 5B). This genotype has not been reported from Saudi Arabia therefore only study sequences were analyzed. Four variable sites were identified in the ON1 genotype with 1 amino acid (position 274) in the analogous region, 2 amino acids (position 298, 304) in the 24 amino acid duplicated region and an amino acid at 310 near the C terminal end of the protein.

The data set (n = 11) for the BA genotype included the prototype sequence, 5 study sequences and 6 Saudi sequences that were reported earlier [41] (S2 Table). The BA genotype revealed the largest number of variable sites (25) in the Saudi strains (Fig 5C). Five variable amino acids (position 216, 218, 219, 223 and 229) were identified in the region prior to the analogous region, 3 sites (position 245, 246 and 247) in the analogous region, 8 sites (position 262, 263, 267, 270, 271, 273, 275 and 278) in the 60-bp duplicated region and 5 sites (position 281, 287, 289, 292, and 293) in the C-terminal end of the G protein. One particular amino acid at 281 position that was just adjacent to the duplicated region was the most variable site of the BA genotype with entropy value of 0.91. Another amino acid at 271 position in the duplicated region also had high entropy value of 0.63. The amino acid at position number 219 had entropy value of 0.45 and was identified as the positive site by selection pressure analysis as well.

### N- and O-linked glycosylation sites

N-linked glycosylation sites were predicted for RSVA genotypes (S1 Table) and RSV B genotypes (S2 Table) with respect to their respective prototype strains. The study NA1 sequences (71 amino acids) had 2 N-linked glycosylation sites at 251 and 294 amino which were conserved among all the Saudi strains. Serine and threonine are potential O-linked sugar acceptors. The study sequences showed 3 to 28 predicted serine and threonine residues with G score of 0.5 to 0.92. One particular sequence (RUH/RSVA/14/14) had 28 potential residues of glycosylation. This particular sequence has 13 residues that were predicted to be most likely to contain O-linked sugars with G score more than 0.8. These include 3 serine (amino acids 234, 250 and 287) and 10 threonine (227, 231, 235, 238, 241, 245, 246, 249, 252 and 253). In addition, to serine and threonine residues, one extensive O-glycosylation motif (KPX—TTKX) was identified in many NA1 strains.

Two N-linked glycosylation sites were identified at 237 and 318 amino acids in the ON1 study sequences (94 amino acids). Both these sites were conserved among all the sequences. The Net-O-Glyc predicted 36–37 serine and threonine residues to be glycosylated with G score of 0.5 to 0.97. Ten- eleven residues had G score more than 0.8 in the analyzed region. Additionally, 10 serine and threonine were identified as potential sugar acceptors in the analogous region i.e the region just before the 24 amino acid duplicated region. These included 6 serine (amino acids 260, 267, 270, 275, 277 and 283) and 4 threonine (amino acid 264, 268, 269 and 281). Furthermore, we identified 10 serine and threonine residues which were potential sugar acceptors in the 24 amino acid duplicated region. These included 5 serine (amino acids 291, 294, 299, 301 and307) and 5 threonine (amino acids 288, 292, 293, 305 and 306). Among these potential glycosylated residues 2 different amino acids at position number 299 (serine) and 307 (serine) were predicted to be most likely to contain O-linked sugars and had G score more than 0.8. All the ON1 genotype strains showed one copy of the extensive O-glycosylation motif.

Two sites at 296 and 310 amino acids were predicted to be potential residues for N-linked glycosylation in the 100 amino acid region for the BA genotype. Both these sites were conserved among all the study strains and is located after the duplicated region. An earlier Saudi strain (Accession number JF714707) reported an additional site at 273 amino acids in the duplicated region. The Net-O-Gly predicted 19 potential serine and threonine residues for O-linked glycosylation with G score of 0.5 to 0.84 in the 100 amino acid region for group BA viruses. Among these potential glycosylated residues, the 3 residues were predicted to be most likely to contain O-linked sugars. These 3 residues at position number 228, 232 and 236 had G score more than 0.8. Interestingly, all these 3 amino acids are located adjacent to the analogous region. However, the duplicated region had 2 threonine (position number 260 and 266) that are the potential sugar acceptors with G score of 0.5. The extensive O-glycosylation motif was absent in the BA genotype.

## Discussion

RSV is an important viral pathogen among hospitalized children in Saudi Arabia [51,52,53,54]. Most of these studies have identified respiratory viruses by conventional methods i.e. immunofluorescence assay/ELISA/culture. However, a few investigations have identified RSV using PCR and real time PCR [41,50,55,56]. But limited data are available on description of RSV groups and their associated genotypes from this region [41,50]. Therefore, regular surveillance of ARI with special reference to RSV is needed to describe the evolutionary pattern of this emerging viral pathogen in this region. Our present investigation describes the prevalence of RSV infections in children in two main hospitals during winter season in Riyadh in 2014. In addition, we have also carried out the molecular characterization of circulating strains of RSV from this region.

RSV mainly affects the children less than 5 years of age. Earlier studies have reported maximum number of cases of RSV in children less than 2 years of age [45,57,58]. Maximum number of cases were recruited in 0–6 months in the present investigation that coincides with higher percentage of RSV infection in this age group as reported earlier [57]. But statistical analysis of age wise distribution of RSV positive cases of less than 2 years of age did not reveal any striking differences (data not shown). Further, more number of male cases with RSV infection were observed as compared to the females that is due to the fact that more number of male patients were recruited in the study. Higher percentage of RSV infection in male children has been reported earlier also [5,57]. Fever and nasal discharge were the most common clinical symptoms observed among the patients. Additional investigations on larger patient groups will provide more comprehensive information on correlation of RSV infection with demographic and clinical manifestations.

RSV was identified in 27% of the samples by RT-PCR. A recent study from Riyadh also reported positivity rate of 22% for RSV by RT-PCR [50,59]. Other investigations from Saudi Arabia have reported RSV in various proportions (7% to 54%) [50,51,52,53,54,55,56,59,60,61,62]. Group A RSV (77%) predominated over group B viruses (23%) during the study period. Similarly the predominance of group A RSV over group B viruses was reported from Riyadh in earlier investigations [50,56,59]. Other geographical regions have also described the higher incidence of group A RSV [4,63,64,65]. Two samples (5.7%) were positive for both the groups of RSV in the present study. Dual infection with both the groups of RSV have already been described from different geographical regions [45,63,66,67,68].

Phylogenetic analysis clustered the group A RSV strains in NA1 and ON1 genotypes. Majority of the study group A strains (82.6%) belonged to the NA1 genotype. The NA1 genotype was recently described in a study from Riyadh [50]. The NA1 study sequences formed two different clusters within this genotype. The first cluster of NA1 study sequences grouped with sequences from Germany, Thailand and Brazil [9,26,63]. The second cluster of NA1 study sequences grouped with sequences from India [20]. The ON1 study sequences with 72bp duplication in the G protein gene clustered with sequences from China, Canada, South Africa, Japan, South Korea, Spain, Germany, USA, Italy and India [2,5,15,18,26,69,70,71,72]. This is the first report of ON1 genotype of RSV from Saudi Arabia. All the group B study sequences belonged to the BA genotype with 60bp duplication in the second hyper variable region of the G protein gene [12]. The BA genotype was also reported from Riyadh in a recent investigation [41]. The BA study sequences clustered with the sequences form Germany, Philippines, Spain, Japan, Argentina, Saudi Arabia, India, South Africa and Brazil [2,6,20,26,32,41,64,73].

Thus we were able to identify two genotypes ON1 and BA, concurrently with duplications in the second hyper variable region of G protein gene. This is the first report of concurrent existence of ON1 and BA genotypes in Saudi Arabia. Interestingly, the ON1 and BA genotypes have been circulating worldwide since last 6 and 17 years respectively [5,12]. The genetic variations in these two genotypes occur due to mutations especially in the duplicated region and changes in the stop codon usage leading to formation of subgroups among themselves [6,7,20,63]. Antigenic variations may occur in RSV due to change in the pattern and frequency of glycosylation [74,75]. The antigenic changes in viruses with duplications may assist in immune evasion thus providing additional advantage to virus resulting in their dispersal to different geographical regions. Alternately, the other characteristic features of the G protein such as attachment may be altered rendering these genotypes more fit than existing viruses [45]. Therefore, we analyzed the genetic diversity in the second hyper variable region of G protein gene of the ON1 and BA genotypes identified during the current investigation by determining the point mutations, positively selected sites and variable sites. In addition, the N- and O-linked glycosylation sites for both the ON1 and BA genotypes were also estimated.

Amino acid analysis of the ON1 and BA study strains revealed several mutations in the second hyper variable region of the G protein gene. Two additional mutations were identified in the duplicated region of both BA and ON1 genotypes which indicates gradual accumulation of mutations over time in the duplicated regions of these genotypes as described earlier [76]. Therefore, it is obvious that evolutionary pressure exists on the duplicated regions suggesting more such genetic variations in the BA and ON1 genotypes in future. Some of these mutations may also affect the glycosylation pattern of the G protein as mentioned below. Further, the selection pressure analysis of the BA and ON1 genotypes revealed low dN/dS ratio indicating purifying selection as described earlier [45]. Additionally, a few positively selected sites have been determined for both the genotypes suggesting stochastic process of evolution [4]. However, one amino acid (position 274) in the ON1 genotype was identified to be under positive selection in the present study. This substitution has been linked with antibody escape of RSV in previous reports [4,27,77]. Further, we were able to identify one codon in the BA genotype (codon 219) under weak positive selection pressure. Additional investigations will determine the effect of these two amino acid mutations (274 in group ON1 and 219 in group BA) on the antigenic attributes of the RSV which may provide evolutionary advantage to these two genotypes.

Shannon entropy analysis determined the variable amino acid sites on the G protein gene for the ON1 and BA genotypes of RSV. Although 4 variable amino acids were identified in the ON1 genotype, 2 of these sites were in the duplicated region. One particular variable amino acid at position 274 in analogous region of the ON1 genotype was also reported to be variable by Shanon entropy from Philippines [3]. This particular amino acid was also identified to be positively selected as mentioned above. Further, the BA genotype showed 25 variable sites with 5 sites in the duplicated region. Two amino acids in the duplicated region of the BA genotype (position 267 and 270) were also reported to be variable by Shannon entropy in a recently published report from Philippines [3]. Thus two different amino acids (position 274 in ON1 and 219 in the BA genotypes) were positively selected and had high entropy value suggesting variation at these two positions. However, the role of these mutations in pathogenesis and viral life cycle of the ON1 and BA genotypes should be investigated in future site directed mutagenesis studies. Additionally, the effect of these mutations in global dispersal and continued circulation of these ON1 and BA genotypes in these regions should also be explored in future investigations.

N- and O-linked glycosylation of the G protein are an important landmark of antigenicity of the virus because it may affect the expression of epitopes by either masking or facilitating the antibody recognition resulting in immune evasion [76]. Ten additional O-linked sites in the 24 amino acid duplicated region in ON1 and 2 additional O-linked sites in the 20 amino acid region in BA genotype were identified in the present study. These additional glycosylation sites in the duplicated region may assist these viruses to evade the host immune response thus giving them evolutionary advantage over other existing non-duplicated group A and B viruses. Thus, taken together the amino acid substitutions and change in pattern of glycosylation in the ON1 and BA genotypes may lead to evasion of host immune response. This may further lead to continuous circulation and global spread of these viruses to new territories [57].

Although limited number of samples was analyzed from a single epidemic season in the present study, we demonstrated presence of three different genotypes of RSV including two genotypes with duplication in the G protein gene from Saudi Arabia. However, the ongoing RSV surveillance in this region will further determine the underlying pattern of evolutionary dynamics of these two emerging genotypes with duplication in G protein gene in Saudi Arabia. We are also carrying out full genome sequencing of selected ON1 and BA strains to provide detailed insight into the evolving trend of these genotypes in this region. Additionally, correlation of concurrent infection with these genotypes and disease severity is another aspect that should also be pursued in future investigations.

## Conclusions

In conclusion, we first time describe the preliminary data on co-circulation of group A and B RSV strains with duplication in G protein gene in Riyadh, Saudi Arabia during winter season of 2014. Two different genotypes of group A viruses (NA1 and ON1) and one genotype of group B RSV (BA) were identified during the study. The description of RSV groups and their associated genotypes will help in designing and implementation of vaccines. This is the first report of ON1 genotype and simultaneous detection of RSV genotypes with duplication in G protein gene from the largest country in Middle East. Additionally, establishing a genetic basis and genotypic delineation in the context of infection caused by viruses with duplication in G protein gene will provide intellectual enrichment on disease burden due to RSV in this region. Further, comprehensive investigations involving larger patient groups in both hospital and community settings will contribute towards the understanding of evolutionary trajectory of these emerging genotypes of RSV with duplication in G protein gene in Saudi Arabia.

## Supporting Information

S1 Table List of RSV-A strains used for phylogenetic analysis, selection pressure, entropy analysis and N- and O-linked glycosylation sites in the present study. (DOCX)

S2 Table List of RSV-B strains used for phylogenetic analysis, selection pressure, entropy analysis and N- and O-linked glycosylation sites in the present study. (DOCX)
