# Supplementary material for: Co-Circulation of 72bp Duplication Group A and 60bp Duplication Group B Respiratory Syncytial Virus (RSV) Strains in Riyadh, Saudi Arabia during 2014
Source: PLoS One. 2016 Nov 11;11(11):e0166145. doi: 10.1371/journal.pone.0166145 (PMC5106011; doi:10.1371/journal.pone.0166145)
Supplement: S1 Table — (DOCX) [file pone.0166145.s001.docx]

**S1 Table: List of RSV-A strains used for phylogenetic analysis, selection pressure, entropy analysis and N- and O-linked glycosylation sites in the present study**

| **GenBank Accession No.** | **Strain name** | **Year of isolation**  **(Country)** | **Genotype** | **Phylogenetic analysis** | **Selection pressure analysis** | **Entropy analysis** | **N-and O- linked glycosylation** |
| --- | --- | --- | --- | --- | --- | --- | --- |
| KU726066 | RUH/RSV A/14/14 | 2014  (Saudi Arabia) | NA1 | √ | √ | √ | √ |
| KU726067 | RUH/RSV A/15/14 | 2014  (Saudi Arabia) | NA1 | √ | √ | √ | √ |
| KU726068 | RUH/RSV A/16/14 | 2014  (Saudi Arabia) | NA1 | √ | √ | √ | √ |
| KU726069 | RUH/RSV A/17/14 | 2014  (Saudi Arabia) | NA1 | √ | √ | √ | √ |
| KU726070 | RUH/RSV A/20/14 | 2014  (Saudi Arabia) | NA1 | √ | √ | √ | √ |
| KU726071 | RUH/RSV A/22/14 | 2014  (Saudi Arabia) | NA1 | √ | √ | √ | √ |
| KU726072 | RUH/RSV A/23/14 | 2014  (Saudi Arabia) | NA1 | √ | √ | √ | √ |
| KU726073 | RUH/RSV A/24/14 | 2014  (Saudi Arabia) | NA1 | √ | √ | √ | √ |
| KU726074 | RUH/RSV A/25/14 | 2014  (Saudi Arabia) | NA1 | √ | √ | √ | √ |
| KU726075 | RUH/RSV A/26/14 | 2014  (Saudi Arabia) | NA1 | √ | √ | √ | √ |
| KU726076 | RUH/RSV A/27/14 | 2014  (Saudi Arabia) | NA1 | √ | √ | √ | √ |
| KU726077 | RUH/RSV A/28/14 | 2014  (Saudi Arabia) | NA1 | √ | √ | √ | √ |
| KU726078 | RUH/RSV A/29/14 | 2014  (Saudi Arabia) | NA1 | √ | √ | √ | √ |
| KU726079 | RUH/RSV A/30/14 | 2014  (Saudi Arabia) | NA1 | √ | √ | √ | √ |
| KU726080 | RUH/RSV A/31/14 | 2014  (Saudi Arabia) | NA1 | √ | √ | √ | √ |
| KU726081 | RUH/RSV A/32/14 | 2014  (Saudi Arabia) | NA1 | √ | √ | √ | √ |
| KU726082 | RUH/RSV A/33/14 | 2014  (Saudi Arabia) | NA1 | √ | √ | √ | √ |
| KU726083 | RUH/RSV A/34/14 | 2014  (Saudi Arabia) | NA1 | √ | √ | √ | √ |
| KU726084 | RUH/RSV A/35/14 | 2014  (Saudi Arabia) | NA1 | √ | √ | √ | √ |
| JF714706 | JF714706/ Saudi Arabia/09 | 2009  (Saudi Arabia) | NA1 | √ | √ | √ | √ |
| JX131638 | JX131638/ Saudi Arabia/08 | 2008  (Saudi Arabia) | NA1 | √ | √ | √ | √ |
| JX131639 | JX131639/ Saudi Arabia/08 | 2008  (Saudi Arabia) | NA1 | √ | √ | √ | √ |
| JX131640 | JX131640/ Saudi Arabia/08 | 2008  (Saudi Arabia) | NA1 | √ | √ | √ | √ |
| JX131642 | JX131642/ Saudi Arabia/09 | 2009  (Saudi Arabia) | NA1 | √ | √ | √ | √ |
| JX131644 | JX131644/ Saudi Arabia/09 | 2009  (Saudi Arabia) | NA1 | √ | √ | √ | √ |
| AB470478 | AB470478/ Japan/04 | 2004  (Japan) | NA1 | √ | √ | √ | √ |
| JX568897 | JX568897/  Brazil/10 | 2010  (Brazil) | NA1 | √ | √ |  |  |
| KC342390 | KC342390/ Thailand/11 | 2011  (Thailand) | NA1 | √ | √ |  |  |
| DQ289605 | DQ289605 /China/04 | 2004  (China) | NA1 | √ | √ |  |  |
| KF246587 | KF246587/  India/09 | 2009  (India) | NA1 | √ | √ |  |  |
| KF246643 | KF246643/  India/12 | 2012  (India) | NA1 | √ | √ |  |  |
| KM508818 | KM508818/ Paraguay/10 | 2010  (Paraguay) | NA1 | √ | √ |  |  |
| JF920049 | JF920049/  USA/07 | 2007  (USA) | NA1 | √ | √ |  |  |
| HQ699276 | HQ699276/ South Korea/10 | 2010  (South Korea) | NA1 | √ | √ |  |  |
| KC476706 | KC476706/ South Africa/10 | 2010  (South Africa) | NA1 | √ | √ |  |  |
| JF907053 | JF907053/  India/09 | 2009  (India) | NA1 | √ | √ |  |  |
| HQ731706 | HQ731706/ United Kingdom/08 | 2008  (United Kingdom) | NA1 | √ | √ |  |  |
| GU550471 | GU550471/ China/08 | 2008  (China) | NA1 | √ | √ |  |  |
| JN257703 | JN257703/  Canada/11 | 2011  (Canada) | NA1 | √ | √ |  |  |
| HQ731694 | HQ731694/ Thailand/06 | 2006  (Thailand) | NA1 | √ | √ |  |  |
| FJ391425 | FJ391425/  Germany/07 | 2007  (Germany) | NA1 | √ | √ |  |  |
| KJ710390 | KJ710390/ Germany/12 | 2012  (Germany) | NA1 | √ | √ |  |  |
| M11486 | M11486/  Australia/61 | 1961  (Australia) | A2 | √ |  |  |  |
| AF233917 | AF233917/ USA/94-95 | 1994-95  (USA) | GA1 | √ |  |  |  |
| AF065257 | AF065257/ USA/94 | 1994  (USA) | GA1 | √ |  |  |  |
| DQ248926 | DQ248926/  India/03 | 2003  (India) | GA2 | √ |  |  |  |
| DQ248928 | DQ248928/  India/04 | 2004  (India) | GA2 | √ |  |  |  |
| AF233913 | AF233913/ USA/94-95 | 1994-95  (USA) | GA3 | √ |  |  |  |
| AF516108 | AF516108/ Uruguay/95 | 1995  (Uruguay) | GA3 | √ |  |  |  |
| AF065254 | AF065254/ USA/93 | 1993  (USA) | GA4 | √ |  |  |  |
| AF233906 | AF233906/ Canada/94-95 | 1994-95  (Canada) | GA5 | √ |  |  |  |
| AF348803 | AF348803/ South Africa/97 | 1997  (South Africa) | GA5 | √ |  |  |  |
| AF233918 | AF233918/ USA/94-95 | 1994-95  (USA) | GA6 | √ |  |  |  |
| EU582173 | EU582173 /Brazil/95 | 1995  Brazil | GA6 | √ |  |  |  |
| AF233907 | AF233907/ Canada/94-95 | 1994-95  (Canada) | GA7 | √ |  |  |  |
| AY667088 | AY667088/ Argentina/  00 | 2000  (Argentina) | GA7 | √ |  |  |  |
| AF348807 | AF348807/ South Africa/98 | 1998  (South Africa) | SAA1 | √ |  |  |  |
| DQ171792 | DQ171792/ New Zealand/01 | 2001  (New Zealand) | SAA1 | √ |  |  |  |
| HQ711628 | HQ711628/ South Africa/06 | 2006  (South Africa) | SAA2 | √ |  |  |  |
| HQ711629 | HQ711629/South Africa/07 | 2007  (South Africa) | SAA2 | √ |  |  |  |
| KC476746 | KC476746/South Africa/09 | 2009  (South Africa) | NA2 | √ |  |  |  |
| FJ391432 | FJ391432/  Germany/07 | 2007  (Germany) | NA2 | √ |  |  |  |
| KU726085 | RUH/RSV A/36/14 | 2014  (Saudi Arabia) | ON1 | √ | √ | √ | √ |
| KU726086 | RUH/RSV A/7/14 | 2014  (Saudi Arabia) | ON1 | √ | √ | √ | √ |
| KU726087 | RUH/RSV A/8/14 | 2014  (Saudi Arabia) | ON1 | √ | √ | √ | √ |
| KU726088 | RUH/RSV A/11/14 | 2014  (Saudi Arabia) | ON1 | √ | √ | √ | √ |
| JN257694 | JN257694/  Canada/11 | 2011  (Canada) | ON1 | √ | √ | √ | √ |
| KM434009 | KM434009/China/13 | 2013  (China) | ON1 | √ | √ |  |  |
| JX885731 | JX885731/  South Africa/12 | 2012  (South Africa) | ON1 | √ | √ |  |  |
| KC559442 | KC559442/  China/12 | 2012  (China) | ON1 | √ | √ |  |  |
| AB761609 | AB761609/  Japan/12 | 2012  (Japan) | ON1 | √ | √ |  |  |
| JX627336 | JX627336/  South Korea/11 | 2011  (South Korea) | ON1 | √ | √ |  |  |
| KM402625 | KM402625/  Spain/14 | 2014  (Spain) | ON1 | √ | √ |  |  |
| JX912364 | JX912364/  Germany/12 | 2012  (Germany) | ON1 | √ | √ |  |  |
| KJ672428 | KJ672428/  USA/13 | 2013  (USA) | ON1 | √ | √ |  |  |
| KC858255 | KC858255/  Italy/13 | 2013  (Italy) | ON1 | √ | √ |  |  |
| AB808773 | AB808773/  Japan/11 | 2011  (Japan) | ON1 | √ | √ |  |  |
| KC731482 | KC731482/  India/11 | 2011  (India) | ON1 | √ | √ |  |  |
