# Supplementary material for: Co-Circulation of 72bp Duplication Group A and 60bp Duplication Group B Respiratory Syncytial Virus (RSV) Strains in Riyadh, Saudi Arabia during 2014
Source: PLoS One. 2016 Nov 11;11(11):e0166145. doi: 10.1371/journal.pone.0166145 (PMC5106011; doi:10.1371/journal.pone.0166145)
Supplement: S2 Table — (DOCX) [file pone.0166145.s002.docx]

**S2 Table: List of RSV-B strains used for phylogenetic analysis, selection pressure, entropy analysis and N- and O-linked glycosylation sites in the present study**

| **GenBank Accession No.** | **Strain name** | **Year of isolation (Country)** | **Genotype** | **Phylogenetic analysis** | **Selection pressure analysis** | **Entropy analysis** | | **N-and O- linked glycosylation** |
| --- | --- | --- | --- | --- | --- | --- | --- | --- |
| KU726061 | RUH/RSV B/2/14 | 2014  (Saudi Arabia) | BA9 | √ | √ | √ | | √ |
| KU726062 | RUH/RSV B/3/14 | 2014  (Saudi Arabia) | BA9 | √ | √ | √ | √ | |
| KU726063 | RUH/RSV B/4/14 | 2014  (Saudi Arabia) | BA9 | √ | √ | √ | | √ |
| KU726064 | RUH/RSV B/5/14 | 2014  (Saudi Arabia) | BA9 | √ | √ | √ | | √ |
| KU726065 | RUH/RSV B/6/14 | 2014  (Saudi Arabia) | BA9 | √ | √ | √ | | √ |
| JF714707 | JF714707/  Saudi Arabia/08 | 2008  (Saudi Arabia) | BA9 | √ | √ | √ | | √ |
| JF714708 | JF714708/  Saudi Arabia/09 | 2009  (Saudi Arabia) | BA9 | √ | √ | √ | | √ |
| KC791694 | KC791694/  Saudi Arabia/08 | 2008  (Saudi Arabia) | BA9 | √ | √ | √ | | √ |
| KC791696 | KC791696/  Saudi Arabia/09 | 2009  (Saudi Arabia) | BA9 | √ | √ | √ | | √ |
| KC791697 | KC791697/  Saudi Arabia/09 | 2009  (Saudi Arabia) | BA9 | √ | √ | √ | | √ |
| KC477028 | KC477028/  South Africa/01 | 2001  (South Africa) | BA9 | √ | √ |  | |  |
| DQ227395 | DQ227395/  Argentina/04 | 2004  (Argentina) | BA9 | √ | √ |  | |  |
| KJ710417 | KJ710417/  Germany/13 | 2013  (Germany) | BA9 | √ | √ |  | |  |
| KF246624 | KF246624/  India/10 | 2010  (India) | BA9 | √ | √ |  | |  |
| KM402707 | KM402707/  Spain/13 | 2013  (Spain) | BA9 | √ | √ |  | |  |
| EU635867 | EU635867/  Brazil/06 | 2006  (Brazil) | BA9 | √ | √ |  | |  |
| KJ710409 | KJ710409/  Germany/13 | 2013  (Germany) | BA9 | √ | √ |  | |  |
| AB603467 | AB603467/  Japan/06 | 2006  (Japan) | BA9 | √ | √ |  | |  |
| HM459881 | HM459881/  Japan/09 | 2009  (Japan) | BA9 | √ | √ |  | |  |
| KF246607 | KF246607/  India/10 | 2010  (India) | BA9 | √ | √ |  | |  |
| AB749694 | AB749694/  Philippines/09 | 2009  (Philippines) | BA9 | √ | √ |  | |  |
| AB749712 | AB749712/  Philippines/10 | 2010  (Philippines) | BA9 | √ | √ |  | |  |
| DQ227364 | DQ227364/Argentina/99 | 1999  (Argentina) | BA1 | √ | √ |  | |  |
| AY751131 | AY751131/  Belgium/99 | 1999  (Belgium) | BA1 | √ | √ |  | |  |
| DQ227373 | DQ227373/  Argentina/02 | 2002  (Argentina) | BA1 | √ | √ |  | |  |
| AY333364 | AY333364/  Argentina/99 | 1999  (Argentina) | BA1 | √ | √ | √ | | √ |
| AY751122 | AY751122/  Belgium/03 | 2003  (Belgium) | BA2 | √ | √ |  | |  |
| DQ227389 | DQ227389/  Argentina/03 | 2003  (Argentina) | BA2 | √ | √ |  | |  |
| AY751119 | AY751119/  Belgium/01 | 2001  (Belgium) | BA2 | √ | √ |  | |  |
| DQ227403 | DQ227403/  Argentina/03 | 2003  (Argentina) | BA3 | √ | √ |  | |  |
| DQ227392 | DQ227392/  Argentina/03 | 2003  (Argentina) | BA3 | √ | √ |  | |  |
| DQ227381 | DQ227381/  Argentina/02 | 2002  (Argentina) | BA3 | √ | √ |  | |  |
| DQ227407 | DQ227407/  Argentina/04 | 2004  (Argentina) | BA4 | √ | √ |  | |  |
| AB603484 | AB603484/  Japan/03 | 2003  (Japan) | BA5 | √ | √ |  | |  |
| AB603483 | AB603483/  Japan/02 | 2002  (Japan) | BA5 | √ | √ |  | |  |
| AY751110 | AY751110/  Belgium/03 | 2003  (Belgium) | BA6 | √ | √ |  | |  |
| AY751117 | AY751117/  Belgium/02 | 2002  (Belgium) | BA6 | √ | √ |  | |  |
| AY751116 | AY751116/  Belgium/01 | 2001  (Belgium) | BA6 | √ | √ |  | |  |
| AB603476 | AB603476/  Japan/05 | 2005  (Japan) | BA7 | √ | √ |  | |  |
| AY751087 | AY751087/  Belgium/03 | 2003  (Belgium) | BA7 | √ | √ |  | |  |
| HM459864 | HM459864/  Japan/05 | 2005  (Japan) | BA7 | √ | √ |  | |  |
| AB470482 | AB470482/  Japan/06 | 2006  (Japan) | BA8 | √ | √ |  | |  |
| AB603478 | AB603478/  Japan/06 | 2006  (Japan) | BA8 | √ | √ |  | |  |
| HM459872 | HM459872/ Japan/06 | 2006  (Japan) | BA8 | √ | √ |  | |  |
| HM459884 | HM459884/ Japan/07 | 2007  (Japan) | BA10 | √ | √ |  | |  |
| FJ490355 | FJ490355/  Thailand/08 | 2008  (Thailand) | BA10 | √ | √ |  | |  |
| JX256976 | JX256976/ Malaysia/09 | 2009  (Malaysia) | BA12 | √ | √ |  | |  |
| JX256977 | JX256977/ Malaysia/09 | 2009  (Malaysia) | BA12 | √ | √ |  | |  |
| KF246586 | KF246586/  India/09 | 2009  () | BA12 | √ | √ |  | |  |
| KC791695 | KC791695/ Saudi Arabia /09 | 2009  (India) | BA12 | √ | √ | √ | | √ |
| M17213 | M17213/  USA/62 | 1962  (USA) | 18537 | √ |  |  | |  |
| AF065250 | AF065250/ USA/90 | 2009  (USA) | GB1 | √ |  |  | |  |
| AY751256 | AY751256/ Belgium/88 | 1988  (Belgium) | GB1 | √ |  |  | |  |
| DQ270232 | DQ270232/ China/98 | 1998  (China) | GB2 | √ |  |  | |  |
| KF246637 | KF246637/  India/12 | 2012  (India) | GB2 | √ |  |  | |  |
| AY672701 | AY672701/ Argentina/01 | 2001  (Argentina) | GB3 | √ |  |  | |  |
| AY672698 | AY672698/ Argentina/99 | 1999  (Argentina) | GB4 | √ |  |  | |  |
| AF233931 | AF233931/ USA/94-95 | 1994-95  (USA) | GB4 | √ |  |  | |  |
| AY751281 | AY751281/ Belgium/84 | 1984  (Belgium) | Gb5 | √ |  |  | |  |
| AY751280 | AY751280/ Belgium/85 | 1985  (Belgium) | Gb5 | √ |  |  | |  |
| AY751237 | AY751237/ Belgium/00 | 2000  (Belgium) | GB6 | √ |  |  | |  |
| AF348825 | AF348825/ South Africa /00 | 2000  (South Africa) | SAB1 | √ |  |  | |  |
| AY524573 | AY524573/ Kenya/02 | 2002  (Kenya) | SAB1 | √ |  |  | |  |
| AF348826 | AF348826/ South Africa/ 98 | 1098  (South Africa) | SAB1 | √ |  |  | |  |
| AF309678 | AF309678/ Mozambique/ 99 | 1999  (Mozambique) | SAB2 | √ |  |  | |  |
| AF348821 | AF348821/ South Africa/ 99 | 1999  (South Africa) | SAB2 | √ |  |  | |  |
| AY488795 | AY488795/ Uruguay/ 99 | 1999  (Uruguay) | SAB3 | √ |  |  | |  |
| AY488800 | AY488800/ Uruguay/99 | 1999  (Uruguay) | SAB3 | √ |  |  | |  |
| JN119987 | JN119987/  Cambodia/  09 | 2009  (Cambodia) | SAB4 | √ |  |  | |  |
| JN120007 | JN120007/  Cambodia/  09 | 2009  (Cambodia) | SAB4 | √ |  |  | |  |
